# Pathway Analysis of Leisure Activity and Cognitive Function in the Long Life Family Study

**DOI:** 10.1093/geroni/igaa057.1618

**Published:** 2020-12-16

**Authors:** Nicole Roth, Paola Sebastiani, Stephanie Cosentino, Nicole Schupf, Thomas Perls, Stacy Andersen

**Affiliations:** 1 Boston University School of Medicine, Boston, Massachusetts, United States; 2 Boston University, Boston, Massachusetts, United States; 3 Columbia University Medical Center, New York, New York, United States

## Abstract

Familial longevity and greater involvement in activities purported to build cognitive reserve (e.g. education, cognitively stimulating leisure activity) have both been associated with better cognitive function in later life, yet little is known about how these protective factors relate with one another. In this work, we modeled the associations among familial longevity, proxies of cognitive reserve, and cognitive function in the Long Life Family Study (LLFS). We assessed cognitive function using a comprehensive battery of neuropsychological tests (i.e. Digit-Spans, California Verbal Learning Test, Rey-Osterrieth Complex Figure, phonemic fluency, category fluency, Word Generation, DKEFS Sorting Test, and logical memory) in a subset of LLFS family members and a referent cohort (N=314, mean age 75.7±14.6 years). To model these associations, we used a series of Bayesian hierarchical regression pathways that incorporate a random effect for family relatedness, adjusted by age and sex. All continuous variables were rescaled and bounded to be approximately between (0,1) in order to standardize regression coefficients and to allow for an asymmetrical beta-distribution. Controlling for education level, age, and sex, referents had greater engagement in late-life cognitive activities compared to LLFS family members, β=0.38 (95% CI: 0.18 to 0.57). In turn, those with higher markers of cognitive reserve exhibited better neuropsychological performance. Despite LLFS family members having lower participation in cognitively stimulating leisure activities, there were no differences between LLFS family members and referents on cognitive test performance. These results suggest long-lived family members may have more unique pathways (i.e. genetic/environmental) that preserve cognition later in life.

